# Trousseau syndrome in a patient with advanced oral squamous cell carcinoma: a case report

**DOI:** 10.1186/s13256-018-1833-6

**Published:** 2019-01-29

**Authors:** Ken-ichi Aoyama, Masashi Tamura, Masahiro Uchibori, Yasuhiro Nakanishi, Toshihiro Arai, Takayuki Aoki, Yuko Osawa, Akihiro Kaneko, Yoshihide Ota

**Affiliations:** 1 Department of Oral and Maxillofacial Surgery, Tokai University School of Medicine, 143 Shimokasuya, Isehara, Kanagawa 259-1193 Japan; 2 Department of Oral and Maxillofacial Surgery, National Hospital Organization Shizuoka Medical Center, 762-1 Nagasawa, Shimizu, Sunto, Shizuoka 411-0905 Japan

**Keywords:** Trousseau syndrome, Oral squamous cell carcinoma, Cancer-associated thrombosis

## Abstract

**Background:**

Trousseau syndrome is known as a variant of cancer-associated thrombosis. Trousseau syndrome commonly occurs in patients with lung or prostate cancer. Hypercoagulability is thought to be initiated by mucins produced by the adenocarcinoma, which react with leukocyte and platelet selectins to form platelet-rich microthrombi. This is the first report of Trousseau syndrome in a patient with oral cancer.

**Case presentation:**

Here, we describe the case of a 61-year-old Japanese man diagnosed as having advanced buccal carcinoma (T4bN2bM1; the right scapula, erector spinae muscles, and the right femur), who experienced aphasia and loss of consciousness. Although magnetic resonance imaging showed cerebral infarction, carotid invasion by the tumor and carotid sheath rupturing, cardiovascular problems, and bacterial infection were not present, which indicated Trousseau syndrome.

**Conclusions:**

Trousseau syndrome in oral cancer is rare, but we must always consider cancer-associated thrombosis in patients with advanced stages of cancer regardless of the primary site of the cancer and take steps to prevent it.

## Background

It is well known that patients with advanced malignant disease are at risk of a hypercoagulable condition, and may develop cancer-associated thrombosis (CAT) [1].

Trousseau syndrome (TS) is a known state of CAT and often occurs in patients with advanced solid cancers [2]. TS is defined as chronic disseminated intravascular coagulation (DIC) associated with non-bacterial thrombotic endocarditis. Recovery is rare in patients with TS and there is no established evidence regarding the effects of anticoagulant treatment on this condition [1, 3]. TS is currently used to describe a hypercoagulation disorder in patients with malignancy, similar to CAT [1, 3]. TS commonly occurs in pulmonary, digestive, gynecology, or urinary cancer [1, 3, 4], and no such condition has been reported in a patient with oral cancer.

Here, we described a case of TS in a patient with buccal squamous cell carcinoma (SCC).

## Case presentation

In 2017, a 61-year-old Japanese man was referred to an oral and maxillofacial surgeon in Tokai University Hospital, Isehara, Japan, because of trismus and general fatigue. He complained of gradually worsening trismus and a painful ulcerated wound in the right buccal mucosa that had failed to heal for the past 6 months. He was on medication for hypertension and had no other specific systemic disease. On physical examination, facial swelling without redness was observed on the middle right side of his face, and trismus was noted (inter-incisor distance was 17 mm). Ulceration was observed in the right buccal mucosa, and an indurated mass could be palpated on the skin of his right cheek. Multiple palpable cervical lymphadenopathies were observed. He underwent workup for suspected malignancy of the buccal mucosa. There were no neurological and cardiologic abnormalities.

**Fig. 1:**
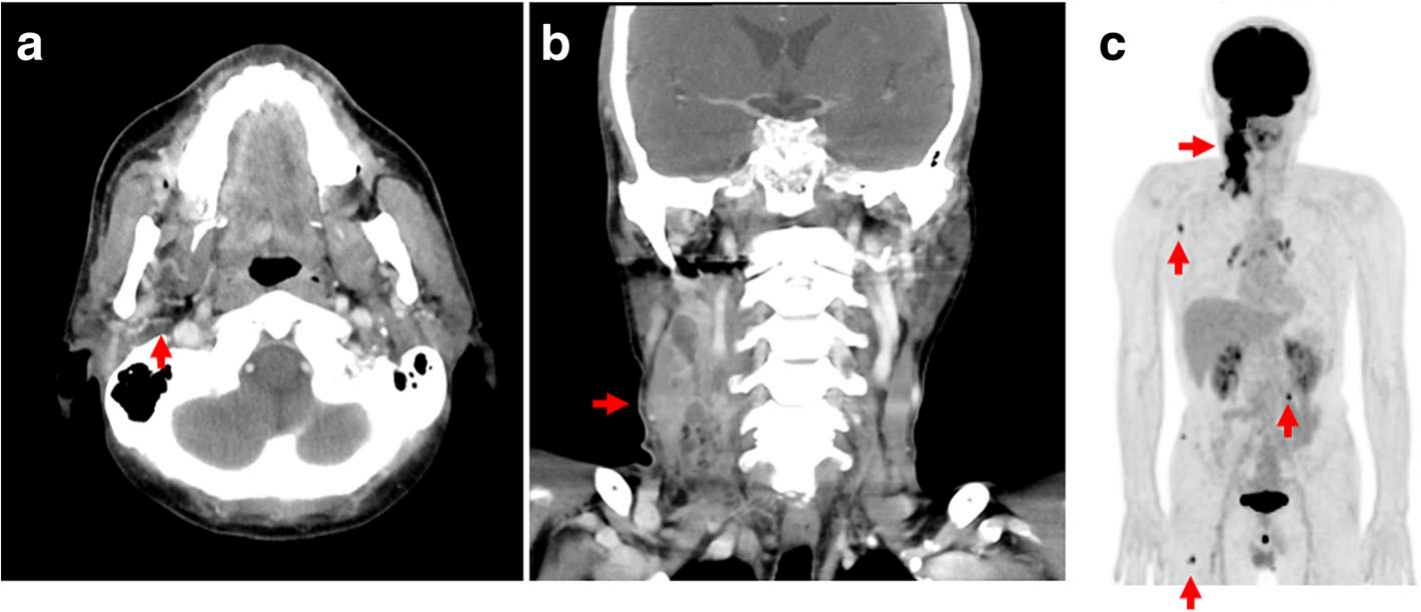
Patient computed tomography scan and positron emission tomography/computed tomography images. **a** and (**b**) Computed tomography showed a mass in the right buccal mucosa (*red arrow*) that extended superiorly to destruct the lateral wall of the maxillary sinus, inferiorly to the retromolar trigone, and laterally to the buccinator muscle and the anterior border of the masseter muscles, with multiple cervical lymph node enlargement. **c** Whole-body ^18^F-fludeoxyglucose positron emission tomography/computed tomography showed increased uptake in multiple lymph nodes in the right cervical area, right scapula and erector spinae muscles, and right femur (*red arrows*)

Computed tomography (CT) showed a mass in the right buccal mucosa that extended superiorly destructing the lateral wall of the maxillary sinus, inferiorly to the retromolar trigone, and laterally to the buccinator and anterior border of the masseter muscles, with multiple cervical lymph node enlargements (Fig. 1a and b). Whole-body ^18^F-fludeoxyglucose (FDG) positron emission tomography (PET)/CT was performed. The PET scan showed increased uptake of FDG in multiple lymph nodes in the right cervical area, scapula and erector spinae muscles, and the right femur (Fig. 1c).

Laboratory tests on admission showed high white blood cell count (13,400 cells/μL) and elevated levels of SCC marker (4.5 ng/mL), but did not show any disorder in other tests including blood coagulation tests and tumor markers: cancer antigen (CA) 19-9, 31 U/ml; and carcinoembryonic antigen (CEA), 1.0 ng/ml.

An incisional biopsy of the right buccal mucosa was performed, which confirmed the diagnosis of SCC. He was given a diagnosis of right buccal carcinoma (T4bN2bM1). Induction chemotherapy was planned, and he was admitted at our hospital. Five days after hospitalization and prior to the initiation of chemotherapy, he experienced aphasia and lost consciousness. He had right hemiparesis with right upper and lower extremities manual muscle test (MMT) grade 0 [5, 6], and his National Institute of Health Stroke Scale (NIHSS) was 19 [7, 8].

**Fig. 2:**
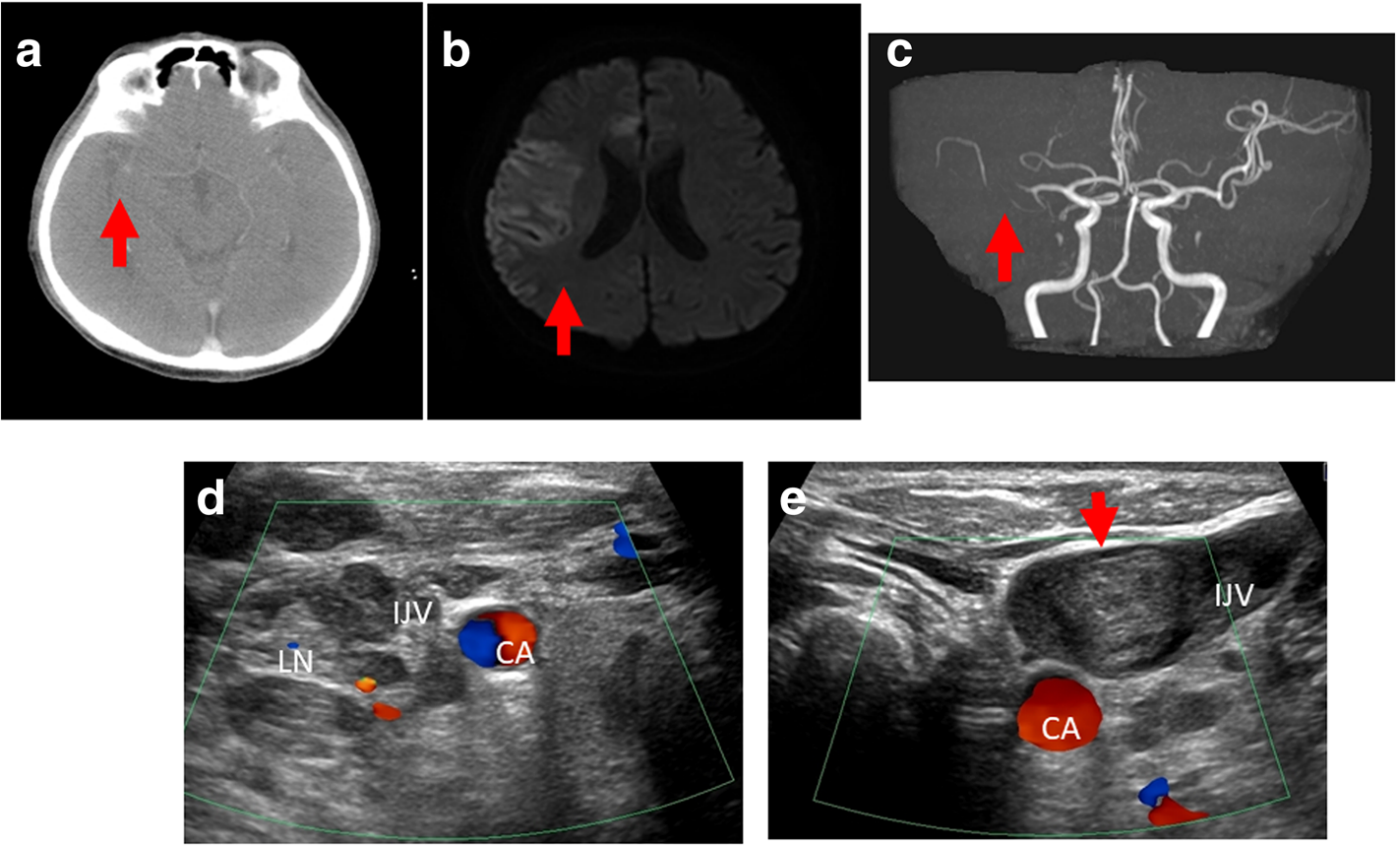
Patient computed tomography scan images after onset of aphasia and loss of consciousness. **a** Scattered hyperdense curvilinear areas (*red arrow*) suggestive of developing petechial hemorrhage in the region of the right middle cerebral artery. **b** Diffusion-weighted image showed a scattered lesion (*red arrow*) affecting the cortical part supplied by the right middle cerebra artery with corresponding deficit. **c** Head magnetic resonance angiography showed attenuated flow-related signal in middle cerebral artery beyond the M1 segment, while its superior division was not visible (*red arrow*). **d** A Doppler ultrasound scan of the neck revealed that the right internal jugular vein was compressed by metastatic lymph nodes. **e** A thrombosis was detected in the left internal jugular vein (*red arrow*). *CA* carotid artery, *IJV* internal jugular vein, *LN* metastatic lymph node

The first set of laboratory tests right after onset revealed a platelet count of 31.1 × 10^4^/μL, a prothrombin time-international normalized ratio (PT-INR) of 1.06, and high levels of fibrinogen degradation product (FDP) at 9.2 μg/ml and D-dimer at 5.4 μg/mL. No marked abnormality was observed on other blood chemistry tests, and the condition did not fulfill the diagnostic criteria for DIC. Brain CT, 30 minutes after the onset of symptoms, showed scattered hyperdense curvilinear areas suggestive of developing petechial hemorrhage in the region of his right middle cerebral artery (MCA) (Fig. 2a). Magnetic resonance imaging (MRI) was performed 100 minutes after the onset of symptoms. Diffusion-weighted image (DWI) showed a scattered lesion affecting the cortical part of the region supplied by his right MCA and perfusion imaging showed corresponding deficit (Fig. 2b). Head magnetic resonance angiography (MRA) showed attenuated flow-related signal in his right MCA region beyond the M1 segment, but its superior division was not visible (Fig. 2c). All imaging findings indicated right MCA infarction. A Doppler ultrasound scan of his neck revealed thrombosis of his left internal jugular vein (IJV), and compression of his right IJV by metastatic lymph nodes (Fig. 2d and e). He was diagnosed as having TS by multifocal cerebral infarction.

Intravenous recombinant tissue plasminogen activator (t-PA) (alteplase 0.6 mg/kg) was administered directly after the MRI scan. Electrocardiogram (ECG), Holter monitoring, echocardiography, and blood culture tests did not show any abnormalities. A head CT on 1, 3, and 7 days after onset showed that the infarction in his right MCA area had not recovered. Seven days after the onset of brain infarction, systemic heparinization was started (PT-INR, 1.5 to 2.0). He did not recover from his cerebral infarction and died 16 days after admission, 21 days after diagnosis, due to pneumonia. A pathological autopsy was not performed as the family did not consent. Family consent was obtained for this case report.

## Discussion

TS was first described in 1865 as migratory superficial thrombophlebitis in patients experiencing cancer [2]. TS commonly occurs in lung (17%), pancreas (10%), colon and rectum (8%), kidneys (8%), and prostate (7%) cancers [4]. This is the first report on TS in a patient with oral cancer or SCC. Recent reports suggested that TS is considered a condition which induces stroke due to the hypercoagulability state associated with malignancy; with non-bacterial and non-circulation thrombotic endocarditis reported as the common causative factor [9–12]. In this case, although we could not carry out transesophageal echocardiography because of trismus, there were no signs of thrombotic or bacterial endocarditis (normal ECG and echocardiography and negative blood culture). In addition, carotid invasion by the tumor and carotid sheath rupturing was ruled out by Doppler ultrasound given the fact that this is the most common cause of head and neck SCC (HNSCC)-associated cerebrovascular attack [13, 14], leading us to the diagnosis of TS in this patient.

TS is described as a chronic disseminated intravascular coagulopathy associated with microangiopathy, verrucous endocarditis, and arterial emboli in patients with cancer, which often occurs in mucin-positive carcinomas of the lung or prostate. Hypercoagulability is thought to be initiated by mucins produced by the adenocarcinoma, which will then react with leukocyte and platelet selectins to form platelet-rich microthrombi [12]. However, the etiology of TS is not known and multiple factors including thromboplastin-like substances, fibrin deposition, direct activation of factor X by tumor proteases, tissue factor, cysteine protease, tumor hypoxia, tumor-induced inflammatory cytokines, are believed to be responsible for this phenomenon in murine models [11, 15–18] of mucinous carcinoma. Although the present case lacks the typical findings of mucin-producing carcinoma, such as intracytoplasmic mucin or extracellular mucin pools, serum tumor markers CA 19-9 and CA-125 were markedly elevated in the tumor, according to immunohistochemical findings.

A recent study, using a large population-based database, indicated that the risk of stroke was significantly higher in patients with HNSCC. However, the risk of stroke in these patients was dependent on age, with the highest rate observed in patients younger than 40 years. The risk was also higher in those patients who had received both radiotherapy and chemotherapy [19]. Our patient did not have any of these risk factors. Thromboprophylaxis in hospitalized patients with cancer is almost universally recommended and two risk scoring systems for venous thromboembolism (VTE) in patients with cancer, namely the Khorana Risk Score (KRS) and Risk Scoring System of CAT (RSSC), are widely used [17, 18, 20, 21]. Although both scoring systems recommend the use of thromboprophylaxis in patients with high or intermediate risk of VTE, both classify patients with head and neck cancer as low risk. This is because both systems are heavily dependent on the site of primary cancer, with gastric and pancreatic cancers scoring the highest on the KRS (2 points), followed by lung, lymphoma, gynecologic, bladder, and testicular cancers (scoring 1); with all other sites, including head and neck, gaining 0 points (Table 1). A similar point system can be observed in RSSC, with myeloma and prostate topping the list with 2 points, lung and gynecologic cancers and sarcoma receiving 1 point, esophagus and breast scoring 1, head and neck and endocrine having a 2-point score, and all other sites are scored as 0 (Table 2). In the present case, the risk of symptomatic VTE was calculated as 0.5–2.1% putting our patient in the intermediate group on the KRS scale and at very low risk on the RSSC. Although both systems recommend anticoagulation in high-risk groups to prevent VTE, patients with HNSCC are rarely categorized as high risk because both systems strongly rely on the primary site of the tumor.

**Table 1:** Khorana Risk Score criteria for assessing venous thromboembolism in patients with cancer

Risk factor	Points		Present patient
Site of primary cancer			
Very high risk (stomach, pancreas)	2		
High risk (lung, lymphatic system, reproductive organs, bladder, testicular)	1		
Low risk (all other sites)	0		0
Other characteristics			
Platelet count ≥350,000/μl	1		
Hemoglobin level < 10 g/dl or use of red cell growth factors	1		
White blood cell count > 11,000/μl	1		1
Body mass index ≥35 kg/m^2^	1		
Risk category	Score (Total points)	Risk of symptomatic VTE	
High risk	≥3	7.1%	
Intermediate risk	1 or 2	2.1%	1
Low risk	0	0.8%	

*VTE* venous thromboembolism

**Table 2:** Risk Scoring System of cancer-associated thrombosis criteria for assessing venous thromboembolism in patients with cancer

Risk factor	Point		Present patient
Age and sex			
40 to 80-year-old female	1		
> 80 years old	−1		
Prior history of VTE	3		
Cancer subtypes			
Low VTE propensity			
Head and neck, endocrine	−2		−2
Esophagus, breast	−1		
High VTE propensity			
Lung, gynecologic, sarcoma, metastasis unknown origin	1		
Myeloma, prostate	2		
Intermediate VTE propensity			
Other cancer subtypes	0		0
Risk category	Score (Total points)	Incidence of VTE	
High risk	3–	8.7%	
Intermediate risk	1–2	1.5%	
Low risk	=0	0.9%	
Very low risk	−4 to −1	0.5%	−2

*VTE* venous thromboembolism

Recovery in TS is slow and there is no established evidence supporting anticoagulant treatment in TS. Controlling the causative tumor and providing immediate systemic anticoagulation are the main steps for the treatment of TS. Systemic heparinization is considered an effective treatment strategy [3, 12, 22, 23].

## Conclusions

Based on our experience with this case, further investigations are required to prevent TS in cases of patients with head and neck carcinoma. If a patient has advanced cancer, there must be discussion concerning whether to use anticoagulation therapy to prevent VTE or not, regardless of the tumor primary site and histological type.

## Abbreviations

CA: Cancer antigen
CAT: Cancer-associated thrombosis
CEA: Carcinoembryonic antigen
CT: Computed tomography
DIC: Disseminated intravascular coagulation
DWI: Diffusion-weighted image
ECG: Electrocardiogram
FDG: ^18^F-fludeoxyglucose
FDP: Fibrinogen degradation product
HNSCC: Head and neck squamous cell carcinoma
IJV: Internal jugular vein
KRS: Khorana Risk Score
MCA: Middle cerebral artery
MMT: Manual muscle test
MRA: Magnetic resonance angiography
MRI: Magnetic resonance imaging
NIHSS: National Institute of Health Stroke Scale
PET: Positron emission tomography
PT-INR: Prothrombin time-international normalized ratio
RSSC: Risk Scoring System of CAT
SCC: Squamous cell carcinoma
t-PA: Tissue plasminogen activator
TS: Trousseau syndrome
VTE: Venous thromboembolism

## References

[1] Evans TR, Mansi JL, Bevan DH. Trousseau’s syndrome in association with ovarian carcinoma. Cancer. 1996;77:2544–9. 10.1002/(SICI)1097-0142(19960615)77:12<2544::AID-CNCR18>3.0.CO;2-S 8640704

[2] Carrier M, Le Gal G, Wells PS, Fergusson D, Ramsay T, Rodger MA. Systematic review: the Trousseau syndrome revisited: should we screen extensively for cancer in patients with venous thromboembolism? Ann Intern Med. 2008;149:323–33. 10.7326/0003-4819-149-5-200809020-00007 18765702

[3] Ikushima S, Ono R, Fukuda K, Sakayori M, Awano N, Kondo K. Trousseau’s syndrome: cancer-associated thrombosis. Jpn J Clin Oncol. 2016;46:204–8. 10.1093/jjco/hyv165 26546690

[4] Sorensen HT, Mellemkjaer L, Olsen JH, Baron JA. Prognosis of cancers associated with venous thromboembolism. N Engl J Med. 2000;343:1846–50. 10.1056/NEJM200012213432504 11117976

[5] Fan E, Ciesla ND, Truong AD, Bhoopathi V, Zeger SL, Needham DM. Inter-rater reliability of manual muscle strength testing in ICU survivors and simulated patients. Intensive Care Med. 2010;36:1038–43. 10.1007/s00134-010-1796-6 PMC2891143 20213068

[6] Compston A. Aids to the investigation of peripheral nerve injuries. Medical Research Council: nerve injuries research committee. His Majesty’s stationery office: 1942; pp. 48 (iii) and 74 figures and 7 diagrams; with aids to the examination of the peripheral nervous system. By Michael O’Brien for the Guarantors of Brain. Saunders Elsevier: 2010; pp. [8] 64 and 94 figures. Brain. 2010;133:2838–44. 10.1093/brain/awq270 20928945

[7] Goldstein LB, Bertels C, Davis JN. Interrater reliability of the NIH stroke scale. Arch Neurol. 1989;46:660–2. 10.1001/archneur.1989.00520420080026 2730378

[8] Brott T, Adams HP Jr, Olinger CP, Marler JR, Barsan WG, Biller J, *et al.* Measurements of acute cerebral infarction: a clinical examination scale. Stroke. 1989;20:864–70. 10.1161/01.str.20.7.864 2749846

[9] Nishino W, Tajima Y, Inoue T, Hayasaka M, Katsu B, Ebihara K, *et al.* Severe vasospasm of the middle cerebral artery after mechanical thrombectomy due to infective endocarditis: an autopsy case. J Stroke Cerebrovasc Dis. 2017;26:e186–8. 10.1016/j.jstrokecerebrovasdis.2017.06.002 28669652

[10] el-Shami K, Griffiths E, Streiff M. Nonbacterial thrombotic endocarditis in cancer patients: pathogenesis, diagnosis, and treatment. Oncologist. 2007;12:518–23. 10.1634/theoncologist.12-5-518 17522239

[11] Akiyama T, Miyamoto Y, Sakamoto Y, Tokunaga R, Kosumi K, Shigaki H, *et al.* Cancer-related multiple brain infarctions caused by Trousseau syndrome in a patient with metastatic colon cancer: a case report. Surg Case Rep. 2016;2:91. 10.1186/s40792-016-0217-7 PMC5011467 27595586

[12] Ladizinski B, Federman DG. Trousseau syndrome. CMAJ. 2013;185:1063. 10.1503/cmaj.121344 PMC3761012 23184841

[13] Conley JJ. Carotid artery surgery in the treatment of tumors of the neck. AMA Arch Otolaryngol. 1957;65:437–46. 10.1001/archotol.1957.03830230013003 13410277

[14] Moore O, Baker HW. Carotid-artery ligation in surgery of the head and neck. Cancer. 1955;8:712–26. 10.1002/1097-0142(1955)8:4<712::aid-cncr2820080414>3.0.co;2-l 13240653

[15] Varki A. Trousseau’s syndrome: multiple definitions and multiple mechanisms. Blood. 2007;110:1723–9. 10.1182/blood-2006-10-053736 PMC1976377 17496204

[16] Kato T, Yasuda K, Iida H, Watanabe A, Fujiuchi Y, Miwa S, Imura J, *et al.* Trousseau’s syndrome caused by bladder cancer producing granulocyte colony-stimulating factor and parathyroid hormone-related protein: a case report. Oncol Lett. 2016;12:4214–8. 10.3892/ol.2016.5152 PMC5104261 27895794

[17] Shao B, Wahrenbrock MG, Yao L, David T, Coughlin SR, Xia L, *et al.* Carcinoma mucins trigger reciprocal activation of platelets and neutrophils in a murine model of Trousseau syndrome. Blood. 2011;118:4015–23. 10.1182/blood-2011-07-368514 PMC3204725 21860019

[18] Wahrenbrock M, Borsig L, Le D, Varki N, Varki A. Selectin-mucin interactions as a probable molecular explanation for the association of Trousseau syndrome with mucinous adenocarcinomas. J Clin Invest. 2003;12:853–62. 10.1172/JCI18882 PMC193671 12975470

[19] Chu CN, Chen SW, Bai LY, Mou CH, Hsu CY, Sung FC. Increase in stroke risk in patients with head and neck cancer: a retrospective cohort study. Br J Cancer. 2011;105:1419–23. 10.1038/bjc.2011.361 PMC3241541 21915128

[20] Mansfield AS, Tafur AJ, Wang CE, Kourelis TV, Wysokinska EM, Yang P. Predictors of active cancer thromboembolic outcomes: validation of the Khorana score among patients with lung cancer. J Thromb Haemost. 2016;14:1773–8. 10.1111/jth.13378 PMC5035574 27273134

[21] Yu YB, Gau JP, Liu CY, Yang MH, Chiang SC, Hsu HC, *et al.* A nation-wide analysis of venous thromboembolism in 497,180 cancer patients with the development and validation of a risk-stratification scoring system. Thromb Haemost. 2012;108:225–35. 10.1160/TH12-01-0010 22534880

[22] Inoue S, Fujita A, Mizowaki T, Uchihashi Y, Kuroda R, Urui S, *et al.* Successful treatment of repeated bilateral middle cerebral artery occlusion by performing mechanical thrombectomy in a patient with Trousseau syndrome. No Shinkei Geka. 2016;44:501–6. 10.11477/mf.1436203318 27270149

[23] Finelli PF, Nouh A. Three-territory DWI acute infarcts: diagnostic value in cancer-associated hypercoagulation stroke (Trousseau syndrome). AJNR Am J Neuroradiol. 2016;37:2033–6. 10.3174/ajnr.A4846 PMC7963789 27365322

